# Bone Position and Ligament Deformations of the Foot From CT Images to Quantify the Influence of Footwear in *ex vivo* Feet

**DOI:** 10.3389/fbioe.2020.00560

**Published:** 2020-06-19

**Authors:** Nicolas Kroupa, Baptiste Pierrat, Woo-Suck Han, Sylvain Grange, Florian Bergandi, Jérōme Molimard

**Affiliations:** ^1^Mines Saint-Etienne, Univ Lyon, Univ Jean Monnet, INSERM, U 1059 Sainbiose, Centre CIS, Saint-Étienne, France; ^2^Centre Hospitalier Universitaire (CHU) de Saint-Étienne, Saint-Étienne, France; ^3^Laboratoire Interuniversitaire de Biologie de la Motricité, Université Jean Monnet, Saint-Étienne, France; ^4^INSERM U1206 Centre de Recherche en Acquisition et Traitement d'Images pour la Sante (CREATIS), Villeurbanne, France

**Keywords:** foot loading, bone registration, ligament model, footwear influence, computed tomography

## Abstract

The mechanical behavior of the foot is often studied through the movement of the segments composing it and not through the movement of each individual bone, preventing an accurate and unambiguous study of soft tissue strains and foot posture. In order to describe the internal behavior of the foot under static load, we present here an original methodology that automatically tracks bone positions and ligament deformations through a series of CT acquisitions for a foot under load. This methodology was evaluated in a limited clinical study based on three cadaveric feet in different static load cases, first performed with bare feet and then with a sports shoe to get first insights on how the shoe influences the foot's behavior in different configurations. A model-based tracking technique using hierarchical distance minimization was implemented to track the position of 28 foot bones for each subject, while a mesh-morphing technique mapped the ligaments from a generic model to the patient-specific model in order to obtain their deformations. Comparison of these measurements between the *ex vivo* loaded bare foot and the shod foot showed evidence that wearing a shoe affects the deformation of specific ligaments, has a significant impact on the relative movement of the bones and alters the posture of the foot skeleton (plantar-dorsal flexion, arch sagging, and forefoot abduction-adduction on the midfoot). The developed method may provide new clinical indicators to guide shoe design and valuable data for detailed foot model validation.

## 1. Introduction

In the moving foot, bones interact with soft tissues such as ligaments, muscles, and fascias, which may undergo large strains. Reliable measurements describing these internal bio-mechanical effects are necessary but difficult to achieve; for example, individual bone movements and deformation of the ligaments of the foot under load are poorly characterized because they are difficult to observe directly. The effect of the shoe has therefore been studied using different types of measurements, including movements of the foot segments, plantar pressures, the user's oxygen demand, associated injuries, and many others (Zipfel and Berger, 2007; Wolf et al., 2008; Hagen and Hennig, 2009; Morio et al., 2009; Fuller et al., 2015; Ferber and Hettinga, 2016); but the impact of the shoe on the internal biomechanical behavior is poorly characterized as there is no available methodology to obtain such measurement. It is likely that the effect of footwear is mainly observed in dynamic and active situations, however understanding the passive and static effect of a shoe on internal structures is a first step toward a better comprehension of the different mechanisms involved.

A study of the internal behavior of the foot under load requires tracking the bone and soft tissue motions. Thus, the development of a method to access each bone movement coupled with accurate ligament positioning allows to describe precisely the internal behavior of the foot and to compare in detail different loading configurations of the foot.

To observe the 3D position of each foot bone, the approaches based on medical images, such as X-rays, are privileged. For dynamic and static cases, 2D images from biplanar fluoroscopy can be used (Ito et al., 2015, 2017; Pansiot and Boyer, 2019). For static case only, images from computed tomography (CT) scanners directly provide 3D information but usually involve a manual processing or interpretation which limits their usage to small sample size (Ferri et al., 2008; Colin et al., 2014; Lintz et al., 2018).

Different algorithms have been developed to segment the bones from CT scans, such as the fuzzy logic approach (Hirano et al., 2000) and graph-cut method (Liu, 2008). They do not systematically provide robust results or still require a significant amount of manual work. When a sequence of different images of volumes evolving though time is considered, an initial segmentation can be associated to a tracking technique in order to determine the movement of each bone between an reference and a current situation. These model-based techniques (Udupa et al., 1998) consist of finding the translation and rotation of a Volume Of Interest (VOI) with minimization of a cost function. For example, in his approach, Liu minimizes an energy function that utilizes both boundaries and region-based informations (Liu et al., 2008). The reference situation can thus be carefully segmented using manual or semi-automatic approaches; then, the processing of successive acquisitions made on the subject will be shorter and more automated.

By combining the tracking of each bone between different situations with a patient-specific representation of connective tissues, an analysis of their deformations can be performed. This patient-specific modeling of ligaments can be done by different approaches. The most manual technique is to interpret the patient's medical images and use anatomical atlases. In multibody models, transfer functions based on interpolating techniques are often used to deform an initial model to fit the patient's anatomy. The transfer function can be of different natures, the simplest being scaling. To deform an initial geometry, also called *source* geometry, into the patient's geometry, called *target* geometry, other more sophisticated methods can be used such as mesh-morphing (Sigal et al., 2008; Grassi et al., 2011).

In this study, we propose an experimental protocol and an associated post-processing methodology to track bone motion and model ligaments in order to describe the influence of the shoe on the position of the bones and the deformation of the ligaments in a restricted static environment with a limited number of *ex vivo* feet; we observe whether bone movement and ligament strain are influenced by wearing a shoe in a passive state to give a first insight into the impact of the shoe on the foot's internal behavior.

## 2. Materials and Methods

### 2.1. Global Strategy

A summary of the entire protocol is given here.

First, a *generic source model* of the foot including the geometry of each bone and ligament attachment points was manually constructed from the Visible Human Project (VHP) images using classic segmentation tools and anatomy books. This model has a high level of details due to the quality of the images from the VHP, which would be inaccessible on X-rays medical images.

Second, a series of CT scans were performed on new patients experimenting different configurations with and without shoes as described in section 2.2.

Third, image processing was performed, starting with the manual segmentation of each bone on the scanner of the barefoot unloaded configuration. Subsequent CT scans in the series were simply thresholded to coarsely separate bone from tissue, without separating each bone individually, resulting in multiple *skeletal configurations*.

Each bone position was identified on the *skeletal configurations* by minimizing the distance between the *manually segmented bones* and the *skeletal configurations* as described in section 2.3.1.

Each bone of the *generic source model* is deformed to fit the *manually segmented bones* by the morphing technique described in section 2.3.2. The interpolation of the deformation places the patient-specific ligament attachment points.

**Figure 1:**
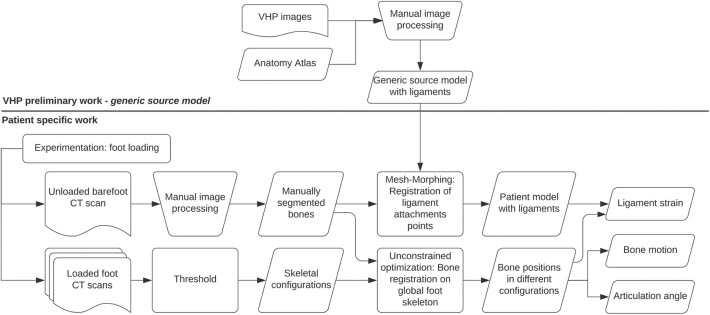
Graphic summary of the protocol.

With this strategy, summarized in the graph shown in the Figure 1, the study of the foot consists of a classic and unique bone segmentation, then automatic placement of ligaments and automatic registration of bones in different scanned configurations, no matter how complex the biomechanical test may be.

### 2.2. Experimentation: Foot Loading

#### 2.2.1. Specimen

The experiments were conducted on a panel of five cadaveric feet coming from different bodies described in Table 1.

**Table 1:** Bodies description.

**Ref**.	**Sex**	**Age (Year)**	**Weight (kg)**
2279	M	86	62
2278	F	85	51
2277	M	94	69
2276	F	91	62
2275	F	74	89

The study was approved by the local Ethics committee (Terre d'Ethique, Saint-Etienne) under the decision IRBN132018/CHUSTE.

When the body arrived, it was directly treated and injected with formaldehyde by the hospital embalmer, then placed in a refrigerator at 4°C.

On average 11 days after the body arrived, it was removed from the refrigerator to separate the lower leg from the upper leg at the knee level; the soft tissue of the lower leg around its upper end is removed, revealing the tibial plateau. The connection between the lower leg so prepared and the compression bench was made by an intermediate mold made of polyurethane resin (PU) cast in a semi-closed cylinder in Polyvinyl Chloride (PVC). This mold links the tibial plateau on one side and a load cell on the other. External anatomical landmarks on the tibial head and the ankle were used in order to align the axis of loading with the axis of the tibia. Once the PU had solidified and therefore the mold was anchored to the anatomical part, the assembly was placed in a freezer at −25°C and remained there for an average of 1 month until 48 h before the tests. Forty-eight hours before the experiments, the foot was placed in a 4°C refrigerator where it remains for 24 h, then was placed at room temperature at 17 degrees for 24 h. Once this defrosting time had elapsed, the foot was attached to the test bench to perform a series of loading tests.

#### 2.2.2. Mechanical Bench

**Figure 2:**
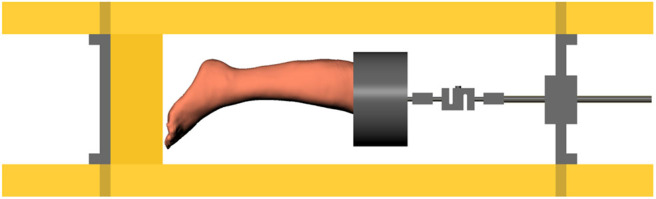
Test bench for loading the foot, while simultaneously measuring the applied force using a load cell.

**Figure 3:**
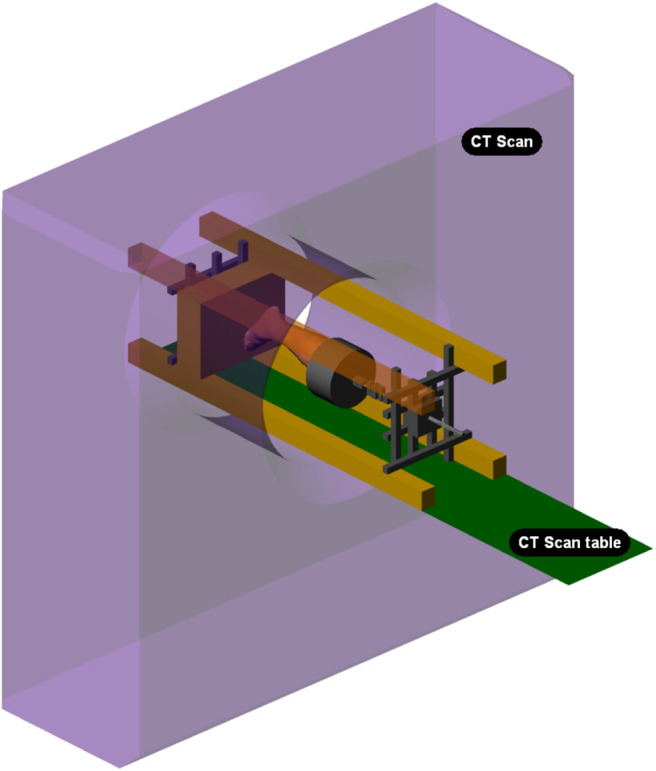
Test bench positioned in the CT scan.

The mechanical loading of the legs was performed in an X-ray tomographic system. A mechanical test bench was developed, its frame being made of two materials: welded steel profiles for parts not exposed to X-rays and treated wood for the columns to reduce X-ray absorption. The load is applied by forcing the axial displacement of the foot in plantar contact with a plane. The displacement is imposed by the axial movement of a threaded shaft. The applied load is continuously recorded using a strain gauge sensor (Phidget 500 Kg S Type Load Cell). The surface plane on which the foot is compressed is adjustable up to 15° in two directions (medial/lateral, antero-posterior) to simulate different foot orientations. The test bench is displayed alone in Figure 2 and placed in the CT scan in Figure 3. The external volume of the bench is 1,500 x 400 x 400 mm^3^.

#### 2.2.3. Experimental Procedure

The five cadaveric feet underwent a series of loading. Each foot was first placed in its natural flat position, then two 15° angular variations were applied: plantar/dorsal flexion and inversion/eversion. In each configuration, the applied load was continuously recorded and the situation was CT scanned punctually at different loads, with and without wearing a shoe, as described in Table 2.

**Table 2:** Tested configuration description.

	**Body weight (BW) loading ratio**			
**Foot orientation**	**Step-0**	**Step-1**	**Step-2**	**Step-3**
Neutral (N)	0	0.1	1.0	1.5
Plantar Flexion (PF)	^a^	0.1	1	^a^
Dorsal Flexion (DF)				
Eversion (EV)	^a^	0.1	1^b^	^b^
Inversion (IV)				

a *Loading not studied because these situations are not encountered by the foot in a classic walking pattern*.

b *Not possible on bare foot due to sliding*.

Three shoes of the same model (Decathlon, Villeneuve-d'Ascq, France) of various sizes were chosen. They are representative of the sports market for occasional runners. The shoes were adapted to each foot size.

**Figure 4:**
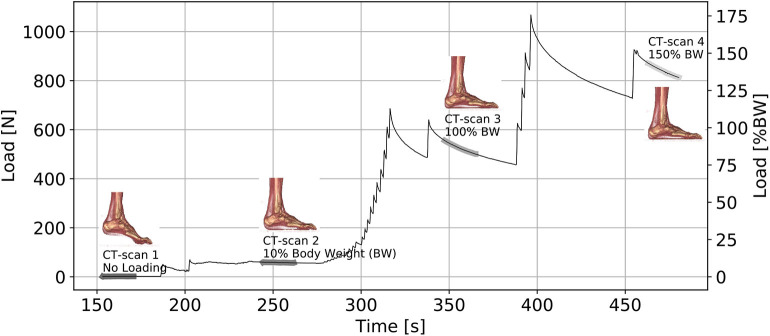
Neutral bare foot acquisition sequence.

A total of 24 scanners, with a resolution of 0.66 x 0.66 x 1.00 mm^3^, were done for each foot. The data acquired for the bare neutral sequence is displayed in Figure 4.

### 2.3. Image Processing

#### 2.3.1. Unconstrained Optimization: Bone Registration on Global Foot Skeleton

The skeleton of the foot, composed of all its bones interconnected, was easily segmented from the 3D images of the CT scan by applying a threshold; it produces a volumetric mask called a *skeleton*.

A classical approach to segment groups of voxels of a threshold, each corresponding to a bone, is to look at their connections and separate the independent groups. Each group can thus be associated to a complete or partial bone part. This simple approach rarely produces good results because the different groups of voxels, each describing a bone, are often interconnected at the joints. Faced with this limitation, the simplest and most manual solution is to remove these voxels creating the interconnections, as no automatic and robust methods are available to our knowledge. Fuzzy logic approaches (Hirano et al., 2000) have not yielded the expected results in our implementation.

To avoid repeating manual operation on all scanners performed on a subject, we used a method based on an optimization process, minimizing the minimum distance between the manually segmented bones in the *initial* position and the *skeleton* mask.

The minimization consists in looking at a rigid transformation that aligns two point clouds such as the Iterative Closest Point (ICP) method (Besl and McKay, 1992; Rusinkiewicz and Levoy, 2001). The unconstrained optimization with Sequential Least SQuare Programming minimization (SLSQP) (Kraft, 1988) provides better results here than the singular value decomposition (SVD) of the corresponding closest point covariance matrix between the two sets as used in ICP. The implementation of SLSQP from the SciPy python package (Virtanen et al., 2019) was used.

The problem is formulated as the following nonlinear unconstrained problem written in the Equation (1).

(1) $$(R,\vec{T})=\text{arg min}\frac{1}{N_{p}}\sum_{i=1}^{N_{p}}{\left\|{\vec{x}}_{i}-(R\cdot {\vec{p}}_{i}+\vec{T})\right\|}^{2}$$

With ***R*** the ***Eulerian*** angles rotation matrix and $\vec{T}$ the *Translation* vector, both composing the rigid transformation. $\vec{p_{i}}$, each point of the initial set of bone points composed of *N*_*p*_ points. $\vec{x_{i}}$ the point of the skeleton closest to $\left(R\cdot \vec{p_{i}}+\vec{T}\right)$.

**Figure 5:**
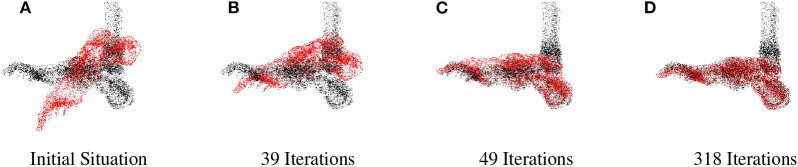
Foot segment minimization. The foot segment is represented with the red point clouds, the loaded *skeleton* is the black dot clouds. **(A)** Initial situation, **(B)** 39 Iterations, **(C)** 49 Iterations, and **(D)** 318 Iterations.

**Figure 6:**
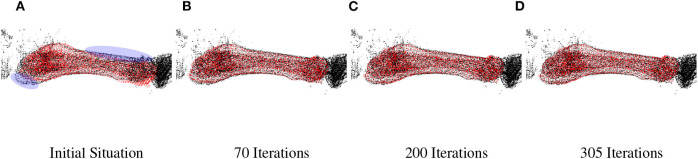
Final minimization on Metatarse 5. The bone is represented with the red point clouds, the loaded *skeleton* is the black point clouds. The initial situation displayed in **(A)** comes from minimizing the previous segment, in blue ellipsoids the non-aligned area are highlighted. **(A)** Initial situation, **(B)** 70 Iterations, **(C)** 200 Iterations, and **(D)** 305 Iterations.

Before individual bone minimization, optimizations of bone segments are performed in a hierarchical fashion in order to obtain acceptable results by avoiding local minima problems. The result of minimization applied to the bone segment defines the initial solution of minimization in the next bone segment. The first treated segment is the entire foot containing all bones except tibia and fibula; its optimization results are presented in Figure 5. The treated segments are described in Table 3. The bone minimization of Metatarse 5 is shown in Figure 6.

**Table 3:** Foot segments description.

**Hierarchy order**	**Segment name**	**Bones included**
1	Foot^a^	All bones except Tibia and Fibula
2	Upper Ankle	Tibia; Fibula
3	Ankle	Calcaneus; Talus
4	MidFoot	Cuneiform Bones; Cuboid
5	Line 1	Metatarsal 1; Medial Sesamoid bones; proximal phalange 1; distal phalange 1
6	Line 2	Metatarsal 2; proximal phalange 2; middle phalange 2; distal phalange 2
7	Line 3	Metatarsal 3; proximal phalange 3; middle phalange 3; distal phalange 3
8	Line 4	Metatarsal 4; proximal phalange 4; middle phalange 4; distal phalange 4
9	Line 5	Metatarsal 5; proximal phalange 5; middle phalange 5; distal phalange 5
10	Metatarse	Metatarsal bones; Sesamoid bones
11	Metatarse1+	Metatarsal 1; Medial Sesamoid bones
12	Proximal phalanges	All proximal phalange bones
13	Middle phalanges	All middle phalange bones
14	Distal phalanges	All distal phalange bones
15	Bone^b^	bone-by-bone minimization from the tibia to distal phalanges

a *Displayed in Figure 5*.

b *Metatarse 5 displayed in Figure 6*.

The tracking of the middle and distal phalanges on the foot *skeleton* may be less robust. This can be increased by subtracting the previous bones found in the point cloud describing the entire *skeleton* during bone-by-bone minimizations. Thus, during the bone-by-bone minimization, the distance will no longer be calculated on the complete *skeleton* but on remaining points only. Good results have been obtained with bone-by-bone minimization by passing from proximal bones to distal bones starting with the bones of the lower leg, then the ankle, midfoot, metatarsus, proximal, middle, and distal phalanges.

#### 2.3.2. Mesh-Morphing: Registration of Ligament Attachments Points

To avoid manual placement of ligaments for each cadaveric foot, the meshes of the *generic source model* bones, whose coordinates of ligament attachment points are known, were deformed toward the geometries of the patients' *target* bones. The *source* model was manually constructed from Visible Human Project (VHP) images and anatomy books.

**Figure 7:**
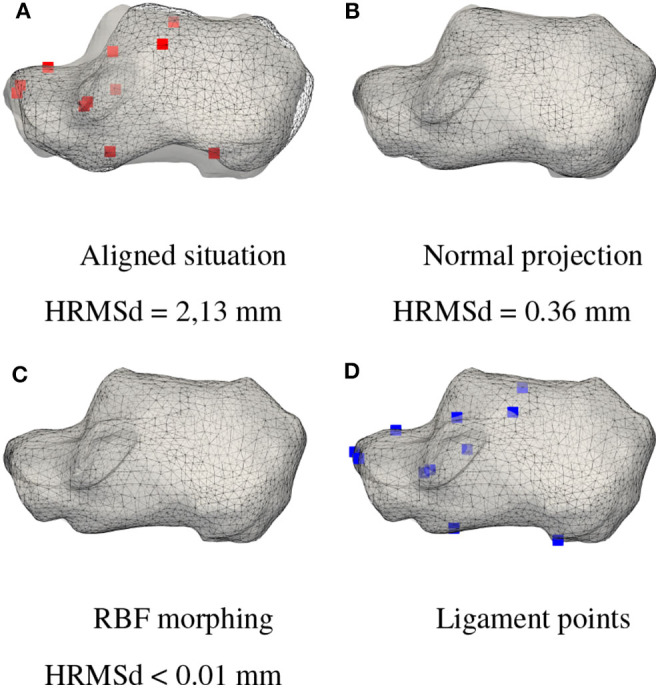
Deformation protocol. Distance between the morphing steps and the *target* is evaluated with the Hausdorff Root Mean Square distance (HRMSd). The patient's *target* mesh is displayed in gray and the *source* bone at different stages of the protocol is displayed in black wireframe. The ligament attachment points of the *source* are displayed in red and those of the patient calculated with the presented protocol are displayed in blue. **(A)** Aligned situation HRMSd = 2, 13 mm, **(B)** Normal projection HRMSd = 0.36 mm, **(C)** RBF morphing HRMSd < 0.01 mm, and **(D)** Ligament points.

Aligning the source and target is an essential step to ensure proper morphing progression. A scaling of the *source* to get closer to the *target* was performed to reduce the difference between the two meshes. The result of the calcaneus bone alignment is shown in Figure 7A.

Once the geometries were aligned, normal projection and smoothing iterations were applied at the *source* to approach the *target*.

Each iteration consisted in the following steps: At time *t*, compute for each points within the polygonal mesh of the current *source* $X(t)=[{\vec{x}}_{i}(t)]$, the normalized normal vectors $N(t)=[{\vec{N}}_{i}(t)]$ pointing toward the nearest *target* surface. Upstream, the nearest distances ${\vec{D}}_{i}=\left\{d_{i}\right\}$ of the *source* nodes with the nearest corresponding point to the *target* were calculated. The index *i* = 1, …, *N*_*i*_ the number of source nodes.

The next positions of the points were calculated with Equation (2)

(2) $$X^{*}(t+1)=[\;{\vec{x}}_{i}(t)+d_{i}w(t){\vec{N}}_{i}(t)\;]$$

With *w*(*t*) a weight factor that can evolve through iterations. In our case, we have chosen a linear rise of this factor up to a maximum value *k*_1_ as described in Equation (3).

(3) $$w(t)=\{\begin{array}{ll} a(1+t) & a(1+t)<w_{1}\\ w_{1} & a(1+t)>w_{1} \end{array}$$

Good results were obtained with a parameter *a* equal to 0.025 and *w*_1_ equal to 1. To avoid the summation of mesh quality degradations on all iterations, we gently smooth with windowed-sinc filter (Taubin et al., 1996), implemented in the Visualization Toolkit (VTK) library (Schroeder et al., 2006), the deformed mesh to obtain the result of the next iteration ***X***(*t* + 1). Iterations were stopped when residual distance was below a threshold. The final iteration of the normal calcaneus projection is shown in Figure 7B.

Once the normal projection is complete, the RBF mesh-morphing can be performed with a variation of the Grassi description (Grassi et al., 2011).

The first step is to establish ***W***(*t*), the weight matrix with only the value of the landmarks positions. The normal distance measurement (ND) (Kim et al., 2012), of each target point on the normally projected source, provides us with the initial position of the marks ${\vec{p}}_{j}$, also written ${\vec{p}}_{l}$ and the final position ${\vec{p}}_{j}^{⋆}$. The index *i* = 1, …, *N*_*i*_ the number of *source* nodes; *j* = *l* = 1, …, *N*_*j*_ the number of *landmark*.

(4) $$D_{land.}(t)=K_{land.}(t)W(t)$$

With ***D***_*land*._(*t*) the landmarks displacements matrix as described in Equation (5) and ***K***_*land*._(*t*) the landmarks RBF matrix as defined in Equation (6).

(5) $$D_{land.}(t)=[\;{\vec{p}}_{j}^{⋆}(t)\;]-[\;{\vec{p}}_{j}(t)\;]$$

(6) $$K_{land.}(t)=[\;k({\vec{p}}_{j}(t),{\vec{p}}_{l}(t))\;]$$

***W***(*t*) is computed with the inversion of ***K***_*land*._(*t*) as in Equation (7)

(7) $$W(t)=D_{land.}(t)K_{land.}^{-1}(t)$$

Once ***W***(*t*) was computed, the RBF matrix of the deformed *source* nodes and landmarks was calculated as equation 8, which is used as an input for the displacement matrix ***D***(*t*) as described in Equation (9). ***D***(*t*) is added to the *source* nodes of the iteration to calculate next node positions as Equation (10)

(8) $$K(t)=[\;k({\vec{x}}_{i}(t),{\vec{p}}_{j}(t))\;]$$

(9) D(t)=K(t)W(t)

(10) $$X^{*}(t+1)=X(t)+D(t)$$

*k* is an inverse multiquadratic RBF function defined in 11.

(11) $$\begin{array}{l} k\left(\vec{x},\vec{p}\right)=\frac{1}{{\left(||\vec{x}-\vec{p}{||}^{2}+||\vec{x}-\vec{p}||c\right)}^{\beta }} \end{array}$$

*c* is the minimum distance of the nearest points between deformed *source* and the *target*. β is described as follows Equation (12)

(12) $$\beta =\{\begin{array}{ll} b*||\vec{x}-\vec{p}|| & b*||\vec{x}-\vec{p}||<k_{1}\\ k_{1} & b*||\vec{x}-\vec{p}||>k_{1} \end{array}$$

Good results were obtained with a parameter *b* equal to 10.0 and *k*_1_ equal to 0.1. To avoid cumulating the mesh degradations of each iteration, a slight smoothing, as described above, can be reused to obtain the result of the iteration ***X***(*t* + 1).

For robust results, 5 morphing iterations were used, increasing the number of landmarks used at each iteration according to their magnitude. The result of calcaneus RBF morphing is shown in Figure 7C.

To register ligament attachment points, displacement vectors between the initial *source* and the morphed *source* are interpolated to calculate the position of the attachment points on the patient bone. The attachment points of the ligaments of the calcaneus are shown in the Figure 7D.

### 2.4. Angle Measurement

**Figure 8:**
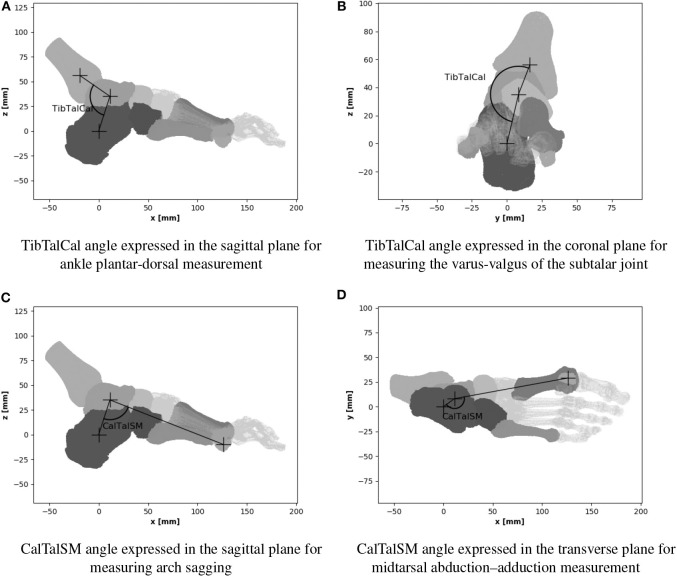
Angle measurement. **(A)** TibTalCal angle expressed in the sagittal plane for ankle plantar-dorsal measurement. **(B)** TibTalCal angle expressed in the coronal plane for measuring the varus-valgus of the subtalar joint. **(C)** CalTalSM angle expressed in the sagittal plane for measuring arch sagging. **(D)** CalTalSM angle expressed in the transverse plane for midtarsal abduction-adduction measurement.

To evaluate the joint angles, two angles between the center of mass of the bones are tracked in the three anatomical planes for each configuration tested. Each angle evaluates the foot position as described in the Table 4 and displayed in Figure 8.

**Table 4:** Angles description.

**Angle name**	**Bones**	**Sagittal**	**Coronal**	**Transverse**
TibTalCal	Tibia; Talus; Calcaneus	Plantar–dorsal flexion (Figure 8A)	Varus–valgus (Figure 8B)	–
CalTalSM	Calcaneus; Talus; Sesamoid Medial	Arch sagging (Figure 8C)	–	Abduction–adduction (Figure 8D)

### 2.5. Statistical Testing

Considering the small size of the sample, it is impossible to prove the normality of the distribution. Therefore, tests between bare and shod feet will be treated using the Wilcoxon signed-rang test as a non-parametric paired test. The level of signitivity is set at *p* = 0.05. Bone motion amplitudes, joint angles and ligament strain were statistically tested between the shod and bare observations.

## 3. Result

### 3.1. Method Results

#### 3.1.1. Experimentation: Feet Loading

We kept three feet out of the five feet tested.

Foot 2276 was discarded because of the considerable difference in load between shod experiments and bare experiments.

Foot 2278 was removed due to a high cadaveric rigidity that prevents foot movement.

The initial scan of the foot 2275, done without load, was performed on the foot in a plantar flexion configuration due to the natural position of this foot, while the feet 2277 and 2279 were initially scanned in the neutral position. The 10% load of body weight (BW) on foot 2279 in plantar flexion could not be achieved due to the length of the test bench being too small for this leg size, forcing a load directly above this ratio.

**Figure 9:**
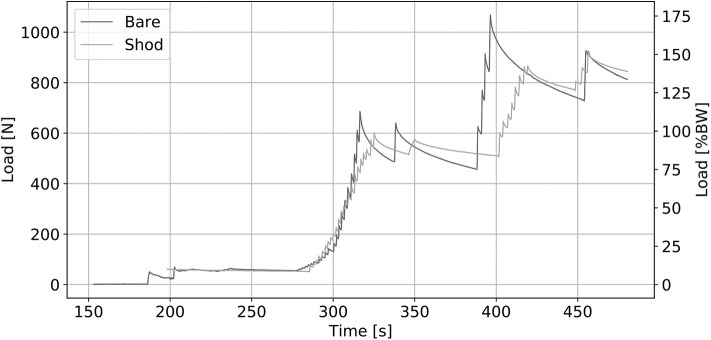
Foot ref. 2279 Loading Sequence on Neutral position.

A greater viscoelastic effect is observed on the bare foot than on the same foot in shoes. Figure 9 shows this difference in viscoelastic effect, especially when the foot is subjected to a load >10% of BW.

#### 3.1.2. Image Processing: Bone Registration on Global Foot Skeleton

The results of bone registration was checked visually and yielded satisfactory positioning. The minimum average distance between each registered bone and the skeleton is 0.28 mm with a standard deviation of 0.09 mm.

#### 3.1.3. Mesh-Morphing of Bones and Registration of Ligament Attachments Points

The Haursdoff distance between the mesh-morphed bone and the target bone is on average 0.06 mm with a standard deviation of 0.09 mm.

**Figure 10:**
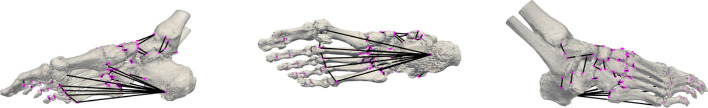
Ligaments positions calculated by the morphing technique.

The calculated ligament positions are shown in Figure 10, a visual inspection validates the positioning of the ligaments identical to the interpretation of anatomical atlases.

### 3.2. Bare vs. Shod Analysis

#### 3.2.1. Bone Motion Amplitude

To first test the interaction between bone translation and footwear; Wilcoxon signed-rank tests were performed on the amplitudes of each bone translation between bare foot and shod foot for each configuration. In 81% of the cases, a significant difference in the amplitude of bone translation was found. The highest differences were observed on dorsal flexion (DF) with a load equal to 0.1 BW. The results are displayed for each foot studied in Figures A1–A4.

The same test is applied to the amplitudes of bone rotation, in 93% of the cases a significant difference is found, again it is in the dorsal flexion configuration with a load equal to 0.1 BW that the greatest difference is observed.

We observed that the difference in bone displacement between shod feet and bare feet, expressed relative to the tibia (see Supplementary Material), are slightly higher for shod feet (mean = 0.04, sd = 0.13). On the contrary, when expressed relative to the calcaneus, which removes the movement of the foot segment, these differences are reversed; the amplitude of the movements of the shod foot is then smaller than that of the bare foot (mean = −1.64 mm, sd = 2.78 mm).

#### 3.2.2. Articulation Angle

Non-parametric statistical tests of angle amplitudes (see Supplementary Material) were performed to test if wearing the shoe affects the chosen joints. The results are presented in Table 5.

**Table 5:** Articulation angle nonparametric tests.

**Parameter**	**Test**	**TibTalCal sagittal**	**TibTalCal coronal**	**CalTalSM sagittal**	**CalTalSM transverse**
Bare/Shod	Wilcoxon	0.067^*^	0.443	5.37e-05^†^	6.29e-05^†^

† *p < 0.05*.

* *p < 0.10*.

Even though the variation is not statistically significant, the TibTalCal angle expressed in the sagittal plane for ankle plantar-dorsal measurement tends to be higher when wearing shoes (*p*-value < 0.10); the median of the angular differences between shod and shod feet is 0.89° resulting from a greater dorsal flexion when wearing the shoe.

No significant difference between shod and barefoot for the TibTalCal angle expressed in the coronal plane measuring the varus-valgus of the subtalar joint was found.

The median difference between the shod and bare CalTalSM angles, expressed in the sagittal plane for measuring arch sagging, is −1.14°, which results in less arch sagging when the footwear is used.

The median of the angle difference between CalTalSM with shoe and bare CalTalSM, expressed in the transverse plane for measuring mid-tarsal abduction-adduction, is −4.46°, resulting in less medial displacement of the forefoot over the midfoot when the shoe is used.

#### 3.2.3. Ligament Deformation

Seventy-four ligaments were modeled and their deformation monitored (see Supplementary Material). The impact of the shoe on ligament deformation was assessed ligament by ligament, 49% had a significant difference in strain between the foot in the shoe and the bare foot.

Certain groups of ligaments see their strain reduced or increased by wearing the shoe; this is the case for the following ligaments:

- Plantar aponeurosis ligaments connecting the medial tuberosity of the calcaneus to the heads of the metatarsals and fingers, in which the median difference between the strain with footwear and the strain without footwear is −1%.
- Long plantar ligament connecting the calcaneus to the proximal epiphysis of the metatarsals 2–5, wherein the median difference between the strain with shoe and the strain without shoe is −0.7%.
- Deep transverse metatarsal ligaments connecting adjacent metatarsal heads, median difference between strain with shoe and strain without shoe is equal to −3%
- Two of the ligaments composing the deltoid ligament complex, the tibiocalcaneal (+2%) and the anterior tibiotalar (−1%) ligaments while no significant difference is found for the posterior tibiotalar and tibionavicular ligaments.
- One of the ligaments composing the bifucarte ligament complex, the calcaneonavicular ligament (+2%) whereas no significant difference is found for the calcaneocuboid ligament.

No significant differences were found for the following ligaments:

- Spring ligament complex
- Plantar and interosseous metatarsal ligaments connecting the proximal epiphysis of the adjacent metatarsals 5 to 2
- Dorsal cuneonavicular ligaments.

## 4. Discussion

The objective of this work was to develop and apply an automated approach to track bone position between different static load cases and provide automated mapping of anatomical components, such as ligaments, to precisely describe the behavior of the foot and use it for overall comparison of the bare foot vs. the shod foot. The combination and automation of these two methods allowed us to make new observations with the tracking of the 3D movements and positions of each bone in static cases associated with the calculation of ligament deformations.

The mesh distance between the global skeleton and each registered bone was used to calculate the registration quality, which is satisfactory. Taking into account that this value in itself is not sufficient to confirm a good record, a visual inspection was also carried out to approve the result. Bone registration errors impact ligament deformation, especially for small ligaments. The evaluation of ligament attachment points is difficult to quantify because we did not compare with the manual placement of these nodes by experienced users. The resulting positioning provides a standard view of the patient's ligaments but does not take into account any specificity of the patient such as ligament injury. The result appears logical and respects the hypothesis formulated on the *source* model although the morphing technique has yet to be tested on severely malformed anatomies.

Finite element, spring and multiple body models are often used to calculate the mechanical properties, deformations, and stresses of anatomical components as well as to simulate foot behavior (Gefen, 2003; Cheung et al., 2005; Sopher et al., 2011; Wei et al., 2011; Chen et al., 2014). Validating such models for a complete foot can be tedious, as there is often a lack of accurate experimental data to compare with simulation results. The measurements obtained with the methods presented here are useful for comparing simulation results with experimental observations and can be used to work on identifying ligament rigidity in the passive state.

The complete digital workflow requires manual processing of one CT scan per patient, automatic placement of ligament and then allows several configurations to be automatically studied. It was performed on a conventional computer workstation (Intel® Core™ i7-6700 CPU @ 3.40GHz × 8 CPU and 16 Go RAM) and lasts about 1 day per foot. A large number of configurations can be easily tested for a given patient, it will not significantly extend the processing time. Large sample sizes of feet can now be treated more easily, including pathological and deformed feet. With this approach, classical clinical indicators such as the foot posture index (FPI-6), the median angle of the longitudinal arch (MLAA) and many others, can be automatically extracted.

As a proof of concept, a very limited clinical study on three cadaveric feet was conducted. Due to the availability of the mobilized CT scan, experiments were conducted first with bare feet and then with feet wearing sports shoes to reduce protocol time by avoiding dressing/undressing repetitions. The manipulation of the anatomical part may have possibly reduced its rigidity; the latter may be less important during the acquisition of the shod foot than during the bare foot tests which can entailed the observations made. Recommendations for future experiments are to randomize the bare and shod trials, increase the load scale and practice preconditioning to reduce the uncertainty of the observations made.

We observed that the foot segments underwent a greater rigid motion during shod experiments. Similar results were reported on walking by comparing barefoot walking and shod walking, as shown by Morio et al. (2009). Nevertheless the authors also found that the plantar/dorsal flexion pattern was not affected by the wearing of shoes, whereas we observed that the amplitude of bone translation is the most influenced by the shoe in dorsal flexion. The type of equipment used, the applied load and the fact that Morio et al. worked with moving feet *in vivo* may explain the difference between their results and our observations.

The relative displacements of the bones in the foot segments are smaller on shod feet than on bare feet.

We interpret these results by considering that the shoe causes rigid movement to the foot segment while restraining the relative movements of these bones. Increased dorsal flexion may be part of the rigid movement of the foot segment when the shoe is worn as shown by the tendency of the TibTalCal angle in the sagittal plane to be higher. The decrease in the relative movement of the bones is also observed by a less pronounced sagging of the plantar arch and a reduced medial displacement of the forefoot when the shoe is worn, it is also confirmed by less deformation of the ligaments, in particular the plantar aponeurosis ligaments.

These results should be interpreted within the main limitation of this study, i.e., passive loading scenarios on cadaveric feet. However, it is a necessary first step in understanding the effect of footwear by decoupling the phenomena involved, as *in-vivo* experiments involve both passive and active effects. Consequently, these results show indications of a passive role of shoes and that their effect under dynamic and active situations should be the focus of future work.

## 5. Conclusion

This paper presents a complete workflow for the study of various cases of foot loading acquired in a scanner, obtaining valuable biomechanical indicators on internal structures, which are difficult to capture experimentally. It is automated and could easily be applied to larger foot samples. This method has been successfully evaluated on three cadaveric feet with a range of applied loads, in different positions, with and without shoes. The combination of accurate 3D bone tracking with a ligament model allowed comparison of bone movement and ligament strain under these various conditions, and provided an interesting insight into the effect of wearing a shoe on these internal structures.

To our knowledge, detailed studies on the moving foot under mechanical load are still lacking in the medical field, but also in other fields (fashion, sport, defense). These studies must comply with *in-silico* clinical requirements, in particular the possibility of treating a representative foot panel and/or developing a patient-specific study. It is therefore necessary to automate the workflow from bone segmentation to mechanical results. The method presented is a step in this direction; it paves the way for the comparison of clinical indicators between different configurations, provides a solid database for the validation of detailed foot models, such as finite element models, which can be exploited for footwear design.

## Data Availability Statement

The original contributions presented in the study are included in the article/Supplementary Material, further inquiries can be directed to the corresponding author/s.

## Ethics Statement

The studies involving human participants were reviewed and approved by Terre d'Ethique, Saint-Etienne IRBN132018/CHUSTE. The patients/participants provided their written informed consent to participate in this study.

## Author Contributions

NK, BP, W-SH, and JM contributed to the design and execution of the research, the analysis of the results, and the writing of the manuscript with the preparation of the body by FB and the acquisitions made under the supervision of SG.

## Conflict of Interest

The authors declare that the research was conducted in the absence of any commercial or financial relationships that could be construed as a potential conflict of interest.
