# Supplementary material for: Bone Position and Ligament Deformations of the Foot From CT Images to Quantify the Influence of Footwear in ex vivo Feet
Source: Front Bioeng Biotechnol. 2020 Jun 19;8:560. doi: 10.3389/fbioe.2020.00560 (PMC7316961; doi:10.3389/fbioe.2020.00560)
Supplement: Supplementary file 1 [file Presentation_1.pdf]

1 Appendix

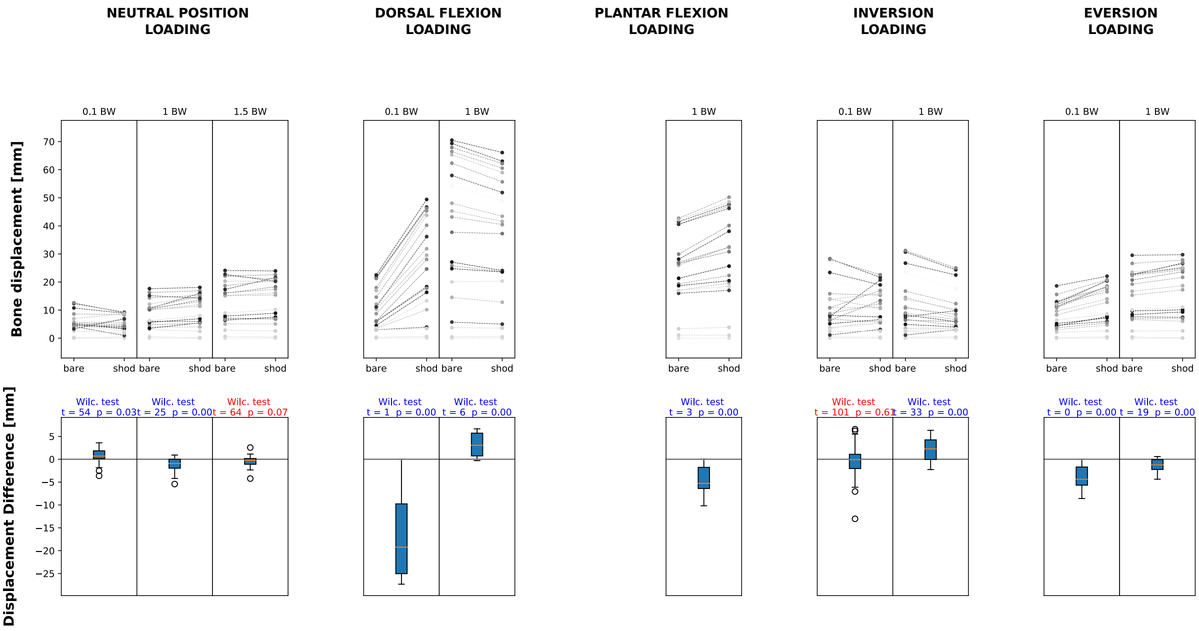

Figure 1: Foot 2279, Wilcoxon signed-rank test on bone translation amplitudes

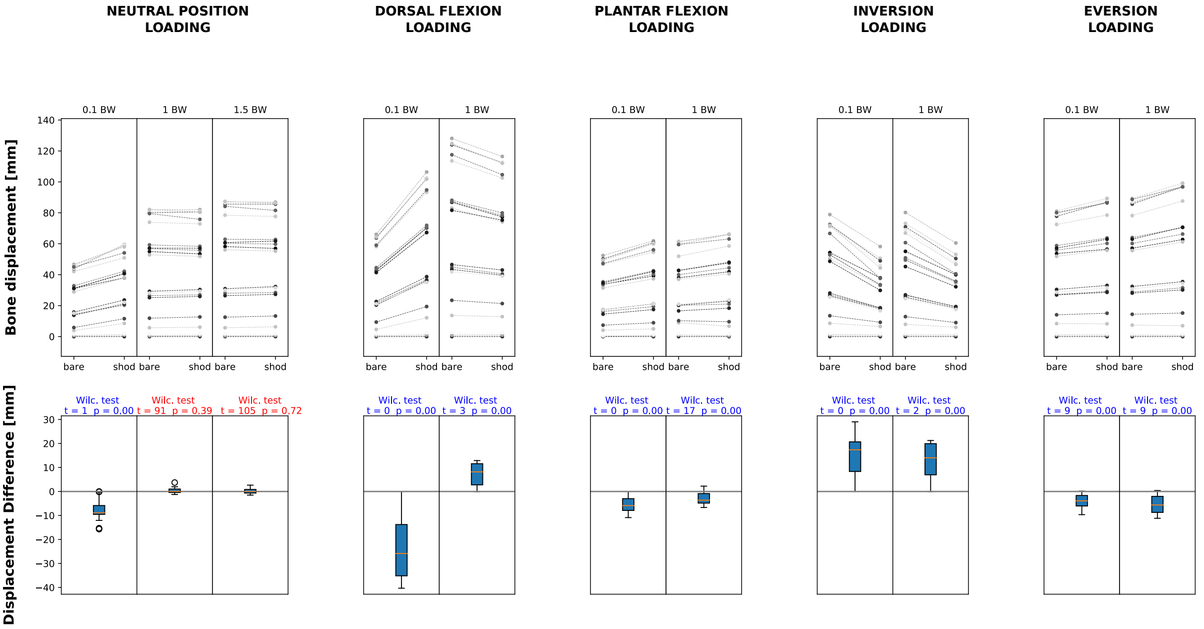

Figure 2: Foot 2277, Wilcoxon signed-rank test on bone translation amplitudes

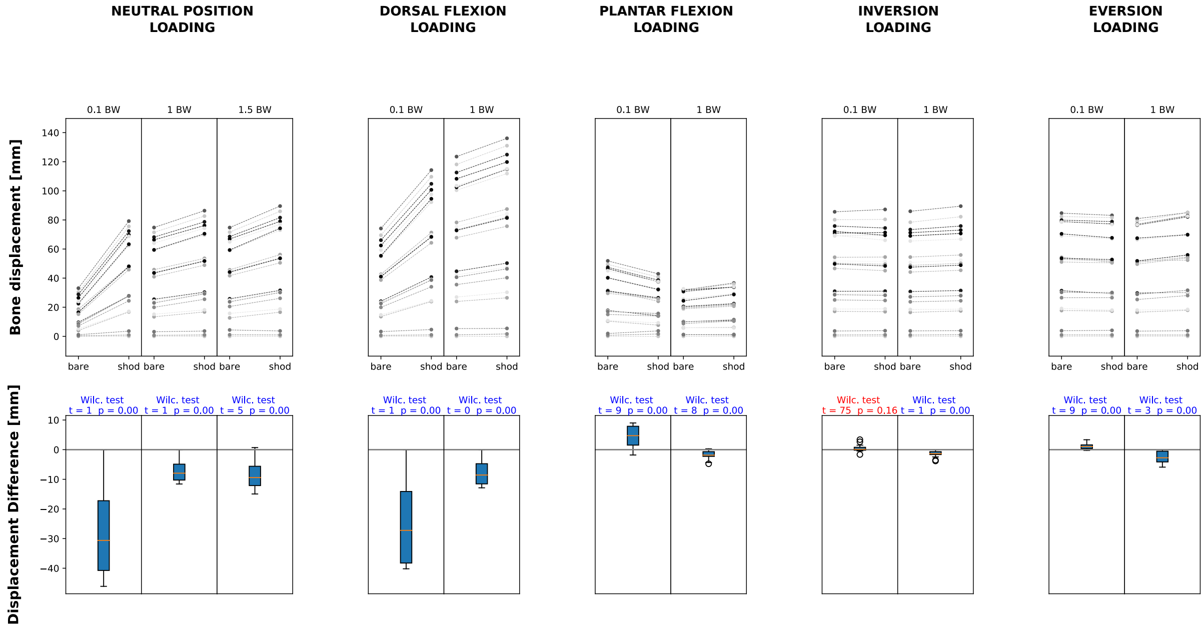

Figure 3: Foot 2275, Wilcoxon signed-rank test on bone translation amplitudes

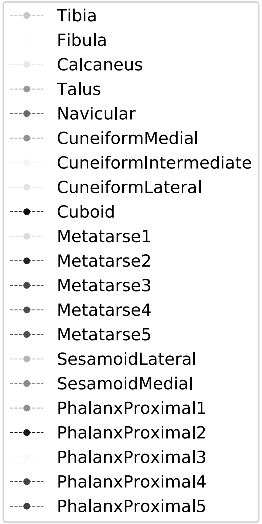

Figure 4: Legend for upper graphs displayed in Figures 1, 2 and 3
